# Left ventricular loading during femoro-axillary veno-arterial ECMO: the role of ECMO–cardiac flow interaction and aortic arch anatomy

**DOI:** 10.1186/s13054-026-06080-z

**Published:** 2026-07-23

**Authors:** Jan Coveliers, Paolo Meani, Simona Celi, Dorela Haxhiademi, Roberto Lorusso

**Affiliations:** 1 Cardio-Thoracic Surgery Department, Maastricht University Medical Centre (MUMC), Maastricht, the Netherlands; 2 Cardiovascular Research Institute Maastricht (CARIM), Maastricht, the Netherlands; 3 Cardiac Surgery Department, University Hospital Antwerp (UZA), Drie Eikenstraat 655, Edegem, BE-2650 Belgium; 4 Faculty of Medicine and Health Sciences, University of Antwerp, Antwerp, Belgium; 5 BioCardioLab, Bioengineering Unit, Fondazione Monasterio, Massa, Italy; 6 Anesthesia and Intensive Care Unit, Ospedale del Cuore, Fondazione Monasterio, Massa, Italy

**Keywords:** Cardiogenic shock, VA-ECMO, Pulmonary capillary wedge pressure, Femoro-axillary, Hemodynamics

To the Editor,

We read with interest the correspondence by Levy and colleagues describing the effect of femoro-axillary (FAx) veno-arterial extracorporeal membrane oxygenation (V-A ECMO) flow on pulmonary capillary wedge pressure (PCWP) [1]. The authors report that increasing ECMO flow from 2 to 4 L/min resulted in minimal changes in PCWP and conclude that FAx ECMO should not be considered superior to femoro-femoral ECMO regarding left ventricular (LV) unloading. While intriguing, this interpretation warrants reconsideration within the broader context of ECMO–heart flow interaction.

Caution is warranted when interpreting PCWP, corresponding to pulmonary artery occlusion pressure (PAOP), as a surrogate of LV loading under V-A ECMO. PCWP and LV end-diastolic pressure are not interchangeable under altered loading conditions [2] and may not reliably reflect left ventricular loading during extracorporeal support, as they represent the net effect of competing preload reduction and afterload increase. This may partly explain the apparent dissociation between ECMO flow and PCWP reported across studies.

Closer examination of the reported data suggests relevant physiological changes despite stable PCWP. Increasing ECMO flow was associated with higher arterial pressures and lower central venous pressures, but also with reduced cardiac index and decreased aortic velocity–time integral, indicating impaired native LV ejection. These findings suggest a shift toward extracorporeal flow dominance, in which stable PCWP may reflect the balance between reduced preload and increased afterload rather than unchanged ventricular loading. This is consistent with physiological and experimental observations demonstrating that left ventricular loading during V-A ECMO is not determined by flow alone, but by the balance between preload reduction and afterload increase, resulting in highly variable and patient-specific responses [3].

Axillary artery cannulation during V-A ECMO is commonly assumed to generate antegrade aortic flow, thereby favoring ventricular unloading and systemic perfusion. However, this assumption likely oversimplifies the interaction between extracorporeal inflow, native cardiac output, and patient-specific aortic arch geometry, including the supra-aortic vessels, which remain insufficiently characterized.

Ventricular loading during V-A ECMO is governed by the interaction between native cardiac output and extracorporeal flow within the arterial system. These competing circulations generate a dynamic interface within the aorta, often referred to as the mixing or “watershed” zone, whose position depends on the balance between native cardiac output and extracorporeal flow. Consequently, LV loading conditions, defined by preload and afterload, are directly influenced by this interaction. Under these circumstances, identical ECMO flow settings may result in markedly different ventricular loading depending on the relative dominance of native and extracorporeal circulation.

Importantly, in approximately 13% of patients, PCWP increased at higher ECMO flows, suggesting that extracorporeal inflow may oppose native ventricular ejection and increase LV afterload rather than facilitate unloading.

Recent computational and experimental investigations suggest that axillary ECMO inflow may generate distinct flow behaviors, including descending-directed, arch-split, or even aortic-valve–directed patterns, highlighting the complex and patient-specific nature of these interactions [4].

Notably, the analysis was limited to patients with a mean arterial pressure above 60 mmHg. Although methodologically understandable, this may have preferentially selected a more hemodynamically stable patient population, potentially closer to a recovery or weaning phase, and excluded those with more pronounced adverse ECMO–heart interactions, particularly in the setting of severely impaired LV function, as suggested in recent clinical observations demonstrating that increasing ECMO flow may significantly alter LV loading conditions depending on baseline ventricular function and timing of support. In such cases, extracorporeal inflow may more markedly oppose extremely weak native ejection, leading to stronger retrograde interaction within the aortic arch.

Cannulation strategy and patient-specific anatomy may further influence these dynamics. All patients underwent right axillary cannulation, reflecting current practice but leaving unanswered whether cannulation side may influence aortic flow interaction and ventricular unloading [5]. Indeed, anatomical variability of the aortic arch may further modulate these effects. Large morphometric analyses demonstrate substantial inter-individual variability, with elongated type III aortic arch (namely, supra-aortic vessels origins located towards the ascending aorta) as the most common pattern (70% over 1.200 thoracic computed tomography of examined adult patients) [5]. In such configurations, the spatial relationship between supra-aortic branches and the ascending aorta may facilitate more retrograde interaction between extracorporeal inflow and native ejection, potentially influencing ventricular afterload and the position of the mixing zone, as shown in the subsequent computational analysis performed with the data obtained by the same computed tomography information and images [4].

Potential effects on cerebral perfusion remain speculative and beyond the scope of this hemodynamic discussion.

These findings highlight that both the assessment of LV loading and the hemodynamic impact of cannulation strategy are highly context dependent. They are determined by the interplay between extracorporeal flow, native cardiac output, and patient-specific vascular anatomy, particularly in the setting of right-sided FAx V-A ECMO. In this context, the hemodynamic impact of axillary cannulation may differ according to cannulation side as well as to supra-aortic vessel/aortic arch and ascending aorta relationship, as suggested by anatomical and computational observations [4, 5]. While requiring further validation, they support a more individualized and anatomical-based approach to cannulation strategy.

Sincerely,

Jan Coveliers.

Paolo Meani.

Simona Celi.

Dorela Haxhiademi.

and Roberto Lorusso.

## Data Availability

Not applicable.

## Abbreviations

ECMO: Extracorporeal membrane oxygenation
FAx: Femoro–axillary
LV: Left ventricle / Left ventricular
PCWP: Pulmonary capillary wedge pressure
V–A ECMO: Veno–arterial extracorporeal membrane oxygenation
